# Treatment of Chronic Posttraumatic Leg Injury Using Autologous Fat Graft

**DOI:** 10.1155/2012/648683

**Published:** 2012-12-19

**Authors:** Fabio Caviggioli, Francesco Maria Klinger, Valeriano Vinci, Guido Cornegliani, Marco Klinger

**Affiliations:** ^1^U.O.C. Chirurgia Plastica, IRCCS Multimedica, Università Degla Studi di Milano, Sesto San Giovanni, 20089 Rozzano, Milano, Italy; ^2^U.O. Chirurgia Plastica 2, Dipartimento di Medicina Traslazionale, Università Degli Studi di Milano e IRCCS Istituto Clinico Humanitas, 20089 Rozzano, Milano, Italy

## Abstract

We present the results obtained in a case of a 20-year-old Caucasian woman with a posttraumatic injury “hard-to-heal” of the left leg treated using autologous fat graft. Considering our experience in treatment of chronic posttraumatic ulcers by autologous fat graft, we decided to use this surgical technique to induce a regenerative effect in this young patient. We have had complete wound closure with only a single surgical procedure after 1 month; after the second intervention of autologous fat graft we observed an improvement in the quality of the scar tissue. The patient satisfaction was excellent. The results were long lasting and remained virtually unchanged after 1 year.

## 1. Introduction

In the last 10 years autologous fat graft has become a surgical technique used in many fields; we applied this technique to scars with improvement of mimic features, skin texture, softness, and thickness; to treat radio damaged tissues, hard-to-heal wound, posttraumatic ulcers, chronic pain syndrome, and in aesthetic surgery [1–12]. We present the results obtained in treatment in a case of posttraumatic ulcer using fat graft.

## 2. Case Reports

An 18-year-old Caucasian woman was refered to our attention for a posttraumatic injury at the left leg; the patient reported to have had a traffic accident two months before the first clinical evaluation, with extensive loss of soft tissue at distal third region of left leg, peroneal nerve injury, and exposure of tendons. 

**Figure 1:**
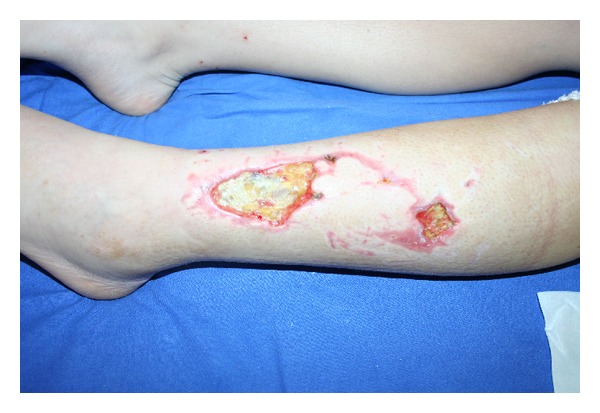
Leg ulcer before any treatment.

During our first clinical evaluation we described two ulcers at distal third level of the left leg that appeared well, cleaned without macroscopic signs of infection. The wounds size was about 7.5 cm to 4 cm in diameter for the first and 3 cm to 3 cm for the second injury (Figure 1). The patient reported that leg ulcers were treated with topical therapies including regular dressing changes, wound cleansers, and advanced dressing with no wound healing improvement or ulcers resolution.

**Figure 2:**
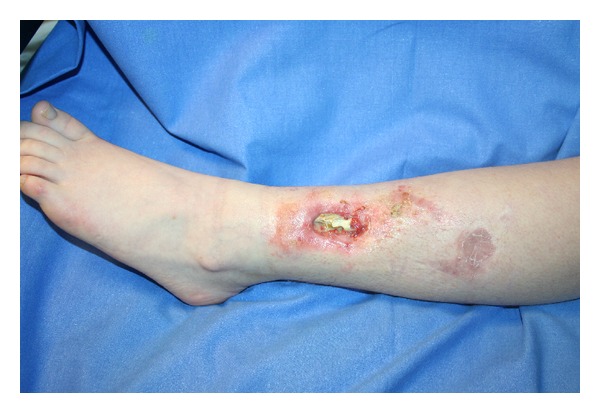
Leg ulcer after Ollier-Thiersch dermoepidermal graft.

Initially we have decided to treat the lesion using an Ollier-Thiersch dermoepidermal graft harvested at the level of the inner face of the ipsilateral thigh. This intervention has resulted in resolution of minor wound and only partial improvement of the major lesion in the left leg; three months after surgical procedure we did not observe spontaneous wound healing improvement (Figure 2). Considering our experience in treatment of chronic post traumatic ulcers by autologous fat graft, we decided to use this surgical technique to induce a regenerative effect in this young patient.

### 2.1. Preoperative Planning

The patient was healthy, without home therapy and nonsmoker.

After standard blood examination and electrocardiography we performed surgical treatment. 

Antibiotic prophylaxis (cefazolin 2 g 30 minutes before the intervention) is administered.

### 2.2. Surgical Procedure

Our goal is to obtaine a full reepithelization of the ulcer using the regenerative potential of autologous fat graft. The surgical procedure is performed under local anesthesia and sedation assisted with sterile technique. Abdomen area represents the donor sites selected for the easy access to the patient in supine position, for the abundant reserves of adipose tissue, and the absence of postoperative outcomes. After the preliminary incision of the skin with a scalpel blade n°11, we proceed to infiltration of the donor areas, at the level of deep subcutaneous tissue, using a blunt cannula filled with a particular anesthetic solution (100 mL saline solution, 10 mL of levobupivacaine 7.5 mg/mL, 20 mL of mepivacaine 10 mg/mL, and 0.5 mL epinephrine 1 mg/mL). Infiltration provides good hemostasis and adequate operative and perioperative analgesic action. Adipose tissue is harvested through the same incision for infiltration of anesthetic solution, with blunt cannulae of 2-3 mm of diameter of variable length (between 15 and 23 cm). The cannula used for sampling is connected with a Luer-lock syringe of 10 cc. The syringe plunger is pulled at the top and secured with a “Backhaus” clamp. This creates, inside the syringe, a slight negative pressure which allows the levy of adipose tissue, while the cannula is advanced and retracted with radial movements inside the donor area. When the syringe is full, it is closed using a Luer-lock cap to prevent loss of content and the syringe plunger is removed. The syringe is now placed in a centrifuge with resterilized containers and adipose tissue is processed following Coleman's technique (i.e., centrifugated at 3,000 rpm for 3 minutes). 

We prefer to harvest fat at the level of deep subcutaneous tissue to avoid risk of skin retraction.

After the centrifugation we obtain, inside the syringe, three distinct layers. The top layer, which is the less dense one, is made of oil derived from the breakdown of fat cells; in the intermediate portion we find adipocytes and stromal-vascular tissue; in the lower level, the denser one, we find damaged blood cells, water, and anesthetic mixture. As the only layer that we need for the therapeutic purpose is the intermediate one, the oily layer is absorbed by tissue strips, and the lower layer is removed by removing the cap from the syringe, in this way permitting the spontaneous drainage of the aqueous portion. Fat is transferred from a 10 mL syringe in a 1 mL syringe Luer-lock that allows precise control of the amount of injected fat and better handling. The adipocyte fraction is injected using an 18-gauge angiographic needle with a snap-on wing (by Cordis, a Johnson & Johnson Company, N.V, 9301 LJ Roden, The Netherlands). 

The adipose tissue fraction has been deposited at the dermal-hypodermal junction in the ulcer's edges with the use of small syringes described above; in addition, fat is grafted into the wound bed. Through the same incision many radiating passages are made, to have fat in different directions according to an ideal form of web to support damaged areas. This technique seems to allow better fat graft survival, to increases rooting and to minimize the possibility of forming cavities filled with triglycerides. The amount of tissue grafted depends on the extension of scar that we need to treat; usually we inject about 1 mL for an area of 2.5 cm^2^ scar surface. In this case we have injected about 5 mL of centrifuged adipose tissue (about 4.5 mg of fat). 

The amount of injected fat at each passage is minimized to avoid irregularities and clusters, which are eventually deleted with digital manipulation after procedure. Injection is performed with retrograde technique leaving a very small space between the injected tissue lines. 

The access incisions in the donor areas are sutured with nylon 3/0. 

A second grafting session of the same volume of graft was performed 6 months later to improve the depth and appearance of the scar tissue.

### 2.3. Dressing

The treated area after surgical debridement was covered with calcium alginate dressing and gauze fixed using paper patches for 1 week and it is recommended to the patient to avoid pressure and friction to limit the displacement of fat infiltration 

At the harvest site level we applied self-adhering open cell foam dressing fixed with an elastic adhesive bandaging which has to be kept in place for 5 days. This type of dressing allow us to prevent hematomas and local fibrosis. 

### 2.4. Postoperative Care

Postoperative paracetamol 1 g/8 h is administered. The patient was monitored for 4 hours after surgery to early identify any local bleeding or systemic symptoms of postoperative discomfort.

### 2.5. Dischargement

The patient is discharged on the same day. Outpatient therapy includes cefixime 400 mg/day for 5 days, paracetamol 500 mg if pain or fever. An ambulatory monitoring was programmed seven days after surgery.

## 3. Results

The dressing is removed 7 days after surgery. The patient has to come to clinical assessment at 1 week, 2 weeks, 1 month, 3, 6, and 12 months.

**Figure 3:**
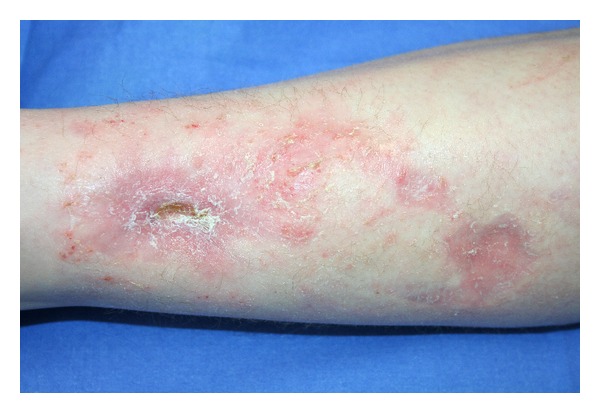
Result 3 months after first grafting session.

**Figure 4:**
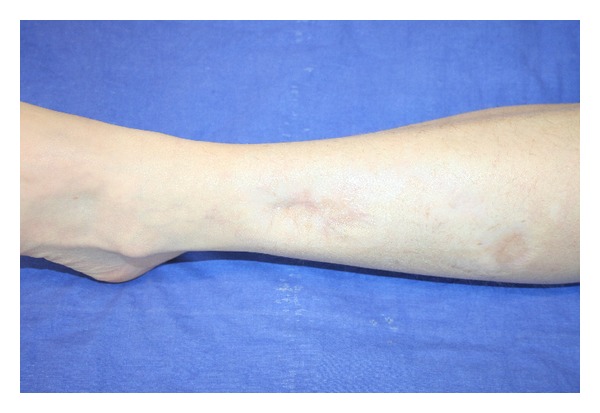
Result 12 months after the first grafting session, 6 months after the second session.

The outcome at 3 months and 12 months after first autologous fat graft can be seen in Figures 3 and 4. We have had complete wound closure with only a single surgical procedure after 1 month; 12 months after first fat graft (6 months after second grafting session) we observed an improvement in the quality of the scar tissue. The patient satisfaction was excellent.

## 4. Discussion

In this paper we showed a complete wound healing in a case of hard-to-heal post traumatic ulcer. The results, already evident at four weeks after surgery, were long lasting and remained virtually unchanged after 1 year.

Since the volume injected by lipostructure is small, even the early results of this procedure can be hardly due to a “filler effect” and suggest that deep biological interactions between transplanted fat and dermal-subdermal structures occur very soon. The rapid improvement of wound healing process and complete recovery of tissue integrity after surgery seems to confirm the regenerative effect of fat graft. Moreover, we have observed an improvement of healed skin quality and elasticity, that appears very similar to normal skin after second fat graft.

Firstly in 2001, Zuk et al. demonstrated the existence of adipose tissue-derived stem cells [13]; then, other studies attempted to discover the operating mechanism of adipose tissue in the regenerative process [14, 15] and the application of its components in different therapies, including regenerative medicine and tissue engineering [16, 17].

The presence of adult mesenchymal stem cells represents an element of great importance for reshaping tissue and increase the quality and elasticity, as observed in the case described.

Although regenerative mechanisms of fat graft are not fully understood, hypoxia could play an important role because it represents an important stimulus for the production of growth factors and neoangiogenesis that facilitating healing.

Lipostructure with Coleman's technique is reported to be a safe, rapid, and effective procedure. 

It is becoming increasingly popular in aesthetic surgery and its range of indications is widening. 

In particular, ulcers treatment by lipostructure is being yielded encouraging results and, in our opinion in the future, will allow to make the application suitable to all types of ulcers (posttraumatic, vascular, and pressure ulcers), especially if no alternative procedure is available.
